# Enhancing ventilation detection during cardiopulmonary resuscitation by filtering chest compression artifact from the capnography waveform

**DOI:** 10.1371/journal.pone.0201565

**Published:** 2018-08-02

**Authors:** Jose Julio Gutiérrez, Mikel Leturiondo, Sofía Ruiz de Gauna, Jesus María Ruiz, Luis Alberto Leturiondo, Digna María González-Otero, Dana Zive, James Knox Russell, Mohamud Daya

**Affiliations:** 1 Department of Communications Engineering, University of the Basque Country (UPV/EHU), Bilbao, Bizkaia, Spain; 2 Department of Emergency Medicine, Oregon Health & Science University (OHSU), Portland, Oregon, United States of America; Azienda Ospedaliero Universitaria Careggi, ITALY

## Abstract

**Background:**

During cardiopulmonary resuscitation (CPR), there is a high incidence of capnograms distorted by chest compression artifact. This phenomenon adversely affects the reliability of automated ventilation detection based on the analysis of the capnography waveform. This study explored the feasibility of several filtering techniques for suppressing the artifact to improve the accuracy of ventilation detection.

**Materials and methods:**

We gathered a database of 232 out-of-hospital cardiac arrest defibrillator recordings containing concurrent capnograms, compression depth and transthoracic impedance signals. Capnograms were classified as non-distorted or distorted by chest compression artifact. All chest compression and ventilation instances were also annotated. Three filtering techniques were explored: a fixed-coefficient (FC) filter, an open-loop (OL) adaptive filter, and a closed-loop (CL) adaptive filter. The improvement in ventilation detection was assessed by comparing the performance of a capnogram-based ventilation detection algorithm with original and filtered capnograms.

**Results:**

Sensitivity and positive predictive value of the ventilation algorithm improved from 91.9%/89.5% to 97.7%/96.5% (FC filter), 97.6%/96.7% (OL), and 97.0%/97.1% (CL) for the distorted capnograms (42% of the whole set). The highest improvement was obtained for the artifact named type III, for which performance improved from 77.8%/74.5% to values above 95.5%/94.5%. In addition, errors in the measurement of ventilation rate decreased and accuracy in the detection of over-ventilation increased with filtered capnograms.

**Conclusions:**

Capnogram-based ventilation detection during CPR was enhanced after suppressing the artifact caused by chest compressions. All filtering approaches performed similarly, so the simplicity of fixed-coefficient filters would take advantage for a practical implementation.

## Introduction

Sudden cardiac arrest is defined as the sudden and often unexpected cessation of the effective contraction of the heart, confirmed by the absence of signs of circulation and breathing [1]. The key techniques during resuscitation of cardiac arrest include airway, breathing and circulation support by means of cardiopulmonary resuscitation (CPR) and defibrillation. In out-of-hospital (OOH) settings, advanced life support (ALS) includes manual defibrillation, advanced airway management, and drug administration during CPR [2, 3].

Capnography is increasingly used by ALS Emergency Medical Services (EMS) systems during the treatment of OOH cardiac arrest [4, 5]. Capnography allows the assessment of the partial pressure of carbon dioxide (CO_2_) in the respiratory gases. The concentration of CO_2_ at the end of the exhalation (ETCO_2_) is considered a surrogate measurement of the pulmonary circulation generated during resuscitation efforts [6]. Customarily, monitoring the capnogram is widely used for guiding ventilation. Excessive ventilation rate has been shown to be frequent and detrimental to the patient during CPR [7–9]. Other uses of capnography in EMS include assessment of the correct positioning of the endotracheal tube [10], monitoring the effectiveness of CPR, identification of restoration of spontaneous circulation [11], and determination of patient prognosis [2, 5, 12].

Quality of the recorded capnogram is essential for a reliable analysis, either visual or automated. However, several authors have reported the appearance of oscillations synchronized with chest compressions distorting capnograms recorded during OOH cardiac arrests [13–17]. Idris et al. specifically reported a high incidence of 70% of distorted OOH capnograms [13]. This phenomenon was not systematically assessed until a recent observational study, in which researchers retrospectively analyzed more than 200 capnograms collected during OOH cardiac arrests [17]. The episodes were classified into distorted (42%) or undistorted (58%), restricting the number of distorted capnograms to those with at least 1 min of distorted ventilations. Three types of artifact were defined according to the location of the oscillations in the respiratory cycle. Finally, the authors reported the negative influence of chest compression artifact in automated detection of ventilations, compromising the reliability of capnogram-based ventilation guidance during CPR.

In this context, we hypothesized that suppressing chest compression artifact from the capnogram was possible using adequate filtering techniques. Filtering would improve the capnogram signal quality and consequently the reliability of automated ventilation detection even in the presence of chest compression oscillations.

The purpose of this study was to explore different filtering techniques to eliminate chest compression artifact from the capnogram. Fixed-coefficient filtering as well as classical adaptive schemas were examined. To assess the filter performance we compared the accuracy of a capnogram-based algorithm for automated detection of ventilations before and after filtering OOH capnograms. We also evaluated the improvement in the measurement of ventilation rate and in the detection of over-ventilation after artifact suppression.

## Chest compression artifact in the capnogram during CPR

**Fig 1:**
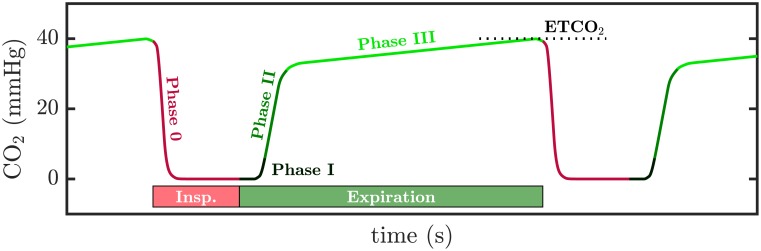
A normal capnogram. The waveform represents the varying CO_2_ levels during the respiratory cycle. Typical segments and phases are named according to [18].

Fig 1 shows the morphology of a normal capnogram, representing the evolution of CO_2_ concentration in the airway with time. Typical intervals and phases are named according to the terminology used by Bhavani-Shankar et al. [18]. During inspiration or phase 0, the airway is filled with CO_2_-free gases, resulting in a rapid decrease of CO_2_ concentration to a zero level that defines the baseline of the capnogram. Expiration comprises three intervals: phase I represents the CO_2_-free gas in anatomical dead space, between the patient’s alveoli and the measurement device; phase II represents the mixture of gases from the anatomical dead space and the alveoli; phase III defines the alveolar plateau, representing the rising of the CO_2_ concentration produced by CO_2_ rich gases coming from the alveoli. The alveolar plateau ends up at a peak level corresponding to the end-tidal CO_2_ concentration (ETCO_2_) [6].

The studies presented in references [14, 15] reported that during CPR, chest compressions generate a fluctuation of little gas volumes that are detected by the capnography sensor, producing oscillations in the capnogram waveform. This artifact has been recently examined in more detail by our research team in a retrospective observational study [17]. We worked with a set of undistorted (clean) and distorted capnograms from patients in OOH cardiac arrest and observed that the artifact appeared as oscillations of varying amplitudes and locations in the capnogram. In that study, we identified three types of artifact, depending on the location of the oscillations: type I, if oscillations appeared in the alveolar plateau; type II, in the baseline; and type III, the most confounding artifact, if the artifact spanned from the plateau to the baseline. No induced oscillations were found in the slopes of phases 0 and II.

**Fig 2:**
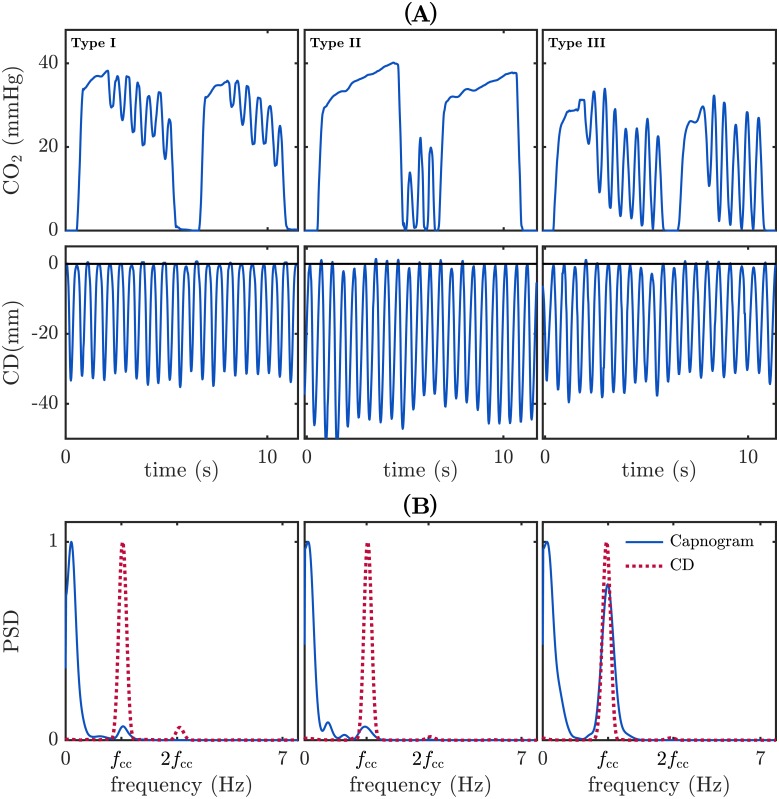
The three different types of observed artifact. (A) Type I, located in the plateau of the capnogram; type II, in the baseline, and type III, spanning from the plateau to the baseline. Each capnogram is depicted with the corresponding CD signal. (B) Power spectral density (PSD) of each capnogram (in solid blue line) and CD signal (in dotted red line). Capnograms present a significant peak at the fundamental frequency of the artifact, *f*_cc_, with highest amplitudes in type III samples.

Fig 2A shows examples of distorted capnograms corresponding to the three observed types of artifact (upper panel). The compression depth (CD) signal depicted below each capnogram shows that the artifact is synchronous with the CD waveform. Fig 2B shows the normalized power spectral density (PSD) estimated for both the capnogram (in solid blue line) and the CD signal (in dotted red line). The PSD of the capnogram presents a low frequency band associated to the ventilation rate (close to 10 per minute in the three examples), and a single peak corresponding to the artifact oscillation frequency. This frequency is exactly the fundamental frequency of the CD signal (*f*_cc_), that is, the chest compression rate. Hence, the artifact presents a sinusoidal characteristic with a fundamental frequency equal to the frequency of the chest compressions.

## Materials and methods

### Data collection and annotation

For this study, data were extracted from OOH cardiac arrest episodes from the Resuscitation Outcomes Consortium (ROC) Epidemiological Cardiac Arrest Registry approved by the Oregon Health & Science University (OHSU) Institutional Review Board (IRB00001736). No patient private data was required for this study. All episodes were recorded using Heartstart MRx monitor-defibrillators (Philips, USA), equipped with real-time CPR feedback technology (Q-CPR) and capnography monitoring using sidestream technology (Microstream, Oridion Systems Ltd, Israel). As the database for this study was the same used in reference [17], we provide here a brief description of the materials. Readers are encouraged to consult the original reference for additional details.

We gathered 232 episodes with the concurrent capnogram, compression depth (CD) signal computed by the Q-CPR technology, and transthoracic impedance (TI) signal acquired from defibrillation pads. Experts participating in the review process manually and visually examined each capnogram and the concurrent CD signal. The CD signal was used as the reference to determine whether chest compressions were provided or not. Episodes were classified as distorted if evident chest compression artifact appeared during more than 1 min of the total chest compression time. Otherwise, episodes were grouped in the clean category. Distorted episodes were then categorized into the artifact categories type I, type II, or type III.

**Fig 3:**
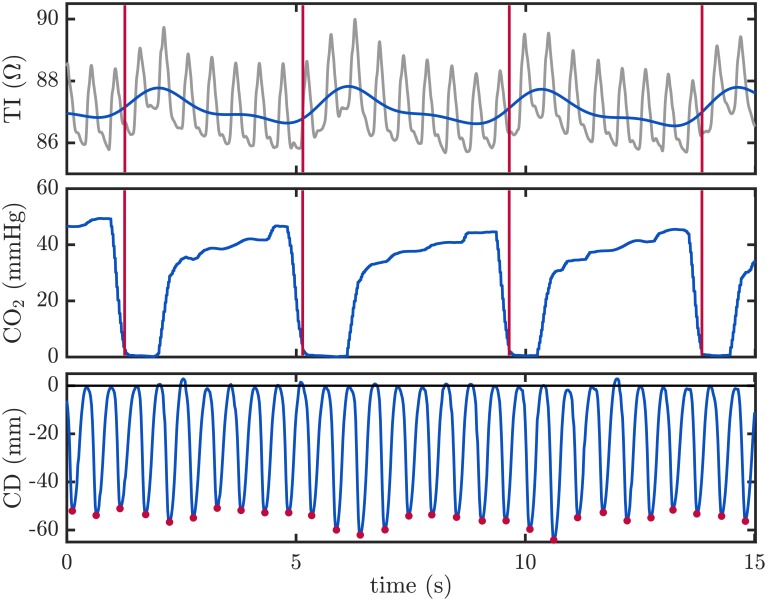
Annotation of ventilations and chest compressions. Ventilations were annotated using the low frequency component of the TI signal (upper panel, in blue), obtained by low-pass filtering the raw TI signal (in gray). Each ventilation was annotated at the rise of a TI fluctuation (red vertical lines). In the capnogram (middle panel), these annotations corresponded to CO2 concentration’s rapid decay to zero. Chest compression instances were annotated in the CD signal (lower panel), and are depicted with red dots corresponding to the instants where the maximum compression depth was achieved.

Ventilations were annotated using the TI signal. Ventilations induce slow fluctuations in the TI signal acquired by defibrillators. TI increases during inspiration due to the increment of the gas volume of the chest and to the longer distance between the electrodes, that produces a decrement in the conductivity [19–21]. The raw TI signal was low-pass filtered to enhance the slow fluctuations caused by ventilations. Experts visually examined the processed TI signal to manually annotate the position of each single ventilation. Fig 3 shows an example of the ventilation annotation. The top panel depicts the raw TI signal in gray with the enhanced low frequency component in blue. Ventilations were annotated at the instant corresponding to a rise in the impedance (vertical red lines). To visually confirm the presence of ventilations the capnogram is depicted in the middle panel. Resulting ventilation annotations were used as the gold standard to evaluate the effectiveness of the proposed filtering techniques. Chest compression instances were annotated at the local minima (Fig 3, bottom panel red dots) corresponding to the maximum depth reached for each chest compression.

### Methods

#### Algorithm for ventilation detection

**Fig 4:**
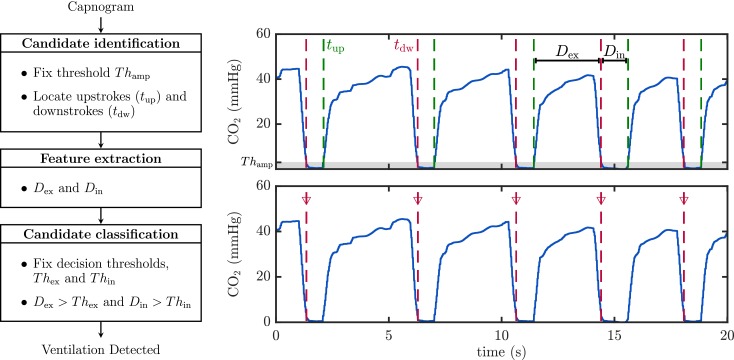
Scheme of the ventilation detector. The algorithm locates upstrokes (*t*_up_) and downstrokes (*t*_dw_) in the capnogram signal (right) applying a fixed amplitude threshold *Th*_amp_. It extracts the duration of the intervals *D*_ex_ and *D*_in_. Finally, fixed duration thresholds *Th*_ex_ and *Th*_in_ are used to discriminate true ventilation from the potential candidates. Detected ventilations are depicted with vertical red dotted lines in the bottom panel.

To assess filtering performance we applied a capnogram-based ventilation detection algorithm before and after artifact suppression [17]. A simplified scheme of the detector is shown in Fig 4. Basically, the algorithm locates series of consecutive upstrokes (*t*_up_) and downstrokes (*t*_dw_) in the capnogram applying an amplitude threshold (*Th*_amp_). Durations between those instants, *D*_ex_ and *D*_in_, are the two features used to classify potential candidates as true ventilations, according to a simple decision tree based on thresholds *Th*_ex_ and *Th*_in_, respectively. Other similar detection algorithms have been previously described in the literature [21].

In the next sections the filtering techniques used for the suppression of the chest compression artifact are presented. We studied three different alternatives: a simple fixed-coefficient filter and two more computationally intensive adaptive filtering techniques.

#### Fixed coefficient (FC) filtering

Observation of the PSD in Fig 2B supports the use of a simple filter with fixed coefficients to suppress the spectral content of the capnogram above 1 Hz (60 cpm). To that end, we implemented a digital infinite impulse response low-pass Butterworth filter.

#### Adaptive filtering

Variability of chest compression rate may affect the efficacy of the FC filter [8, 9, 22, 23]. Adaptive techniques in which the filter parameters are adjusted in time according to the varying characteristics of the artifact could be a suitable solution. In the literature, adaptive filtering has been extensively used for the suppression of the artifact induced by chest compressions in the electrocardiogram recorded by defibrillators during CPR [24–28].

In this study, we designed two different adaptive filtering configurations, an open-loop and a closed-loop adaptive filter [29]. Details of the adaptive filters are addressed in the supporting information S1 Appendix.

**Fig 5:**
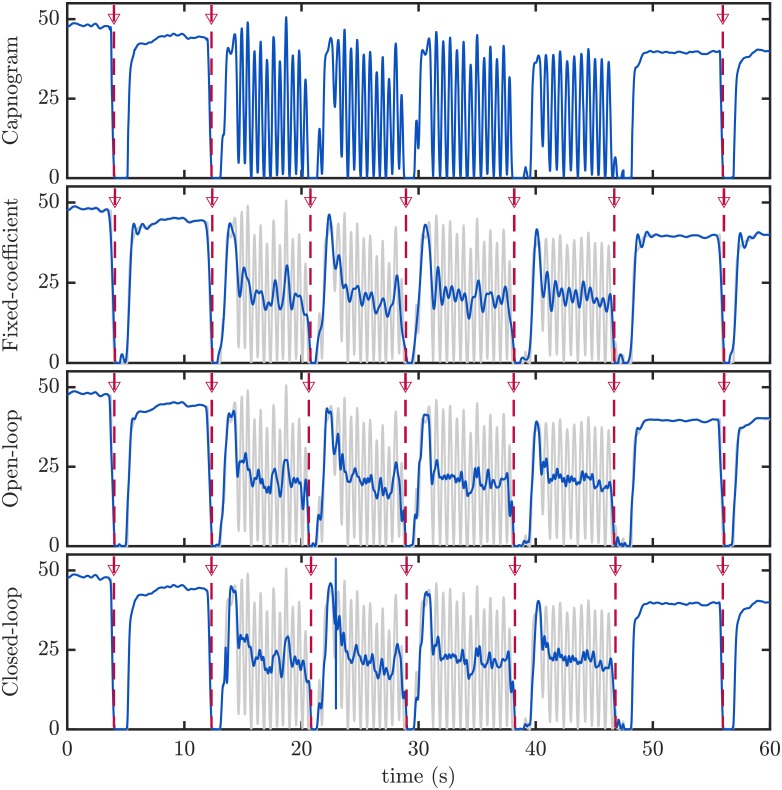
Examples of filtering performance. Original capnogram with clean and distorted respiration cycles (top panel). Detected ventilations are depicted with vertical lines. Distorted ventilations could not be detected by the algorithm. Lower panels show the filtered capnogram (in blue) superimposed to the original capnogram (in gray), for the three filtering alternatives. Detected ventilations are depicted with vertical red dashed lines. In this example, all ventilations were correctly detected after filtering.

Fig 5 illustrates the performance of the filters. The three filtering techniques were applied to the same capnogram (top panel), and the resulting filtered waveforms are depicted in the lower panels (blue line) superimposed on the original capnogram (gray line). Ventilations detected before and after filtering are marked with vertical red dotted lines. Ventilations with chest compression artifact (the four consecutive ventilations in the center of the tracing) were not detected in the original capnogram, but they were successfully identified after artifact cancellation.

### Data analysis and performance evaluation

Ventilation annotations in the database constituted the gold-standard used to evaluate the performance of the automated ventilation detection algorithm applied to the original and to the filtered capnograms. The reliability of the proposed filtering techniques was assessed by comparing the sensitivity (Se) and positive predictive value (PPV) of the ventilation detector before and after filtering. Se was defined as the proportion of annotated ventilations that were correctly detected by the algorithm and PPV was the proportion of detected ventilations that were true ventilations.

Filter parameters were optimized for all filtering strategies with a training subset of 15 clean and 15 distorted capnograms. Optimization criteria was maximum Se for a minimum PPV of 95%. Filter performance was reported for the remaining 202 episodes comprising the test subset.

For each episode in the whole set, we computed the number of ventilations provided every minute (ventilation rate), using a 1 minute sliding window with an overlap factor of 1/6, i.e. the ventilation rate value was updated every 10 s. We compared the ventilation rate measurements computed from the estimated ventilations before and after filtering with those computed from the gold-standard ventilations.

We also tested the accuracy in the detection of over-ventilation, defined as a ventilation rate greater than 10 per minute. This value was selected according to the general recommendation in current resuscitation guidelines [2, 3]. For that purpose, Se was defined as the proportion of annotated over-ventilation intervals that were detected by the algorithm, and PPV as the proportion of true over-ventilation instances among all the over-ventilation alarms provided by the algorithm.

## Results

Table 1 shows a summary of the episodes included in the study. Mean (±standard deviation) duration of the episodes was 31 (±10) min. Airway types were endotracheal tube (ETT) in 64.2%, supraglottic airway (SGA) in 31.7%, and bag-valve-mask (BVM) in 0.03% of the episodes. Distorted episodes comprised 42.2% of the whole set. Type I artifact was annotated in 48%, type II in 21% and type III in 31% of the distorted episodes. A total of 52654 ventilations were annotated, with a mean of 224 (±115) ventilations per episode. A total of 532597 chest compressions were annotated, with a mean of 2296 (±1230) per episode. Mean chest compression rate was 114.0 (±14.4) compressions per minute.

**Table 1:** Characteristics of the episodes included in the study. Values are expressed as mean (±standard deviation).

Group	Episodes	Ventilation type				Duration (min)	Ventilations	Compressions
		BVM	ETT	SGA	NA			
**Total**	232	7	149	73	3	31 (±10)	224 (±115)	2296 (±1230)
**Clean**	134	7	90	35	2	30 (±8)	227 (±124)	1994 (±1247)
**Distorted**	98	0	59	38	1	32 (±12)	221 (±102)	2708 (±1084)
**Type I**	47	0	19	28	0	31 (±7)	212 (±105)	2893 (±1089)
**Type II**	21	0	15	6	0	29 (±6)	249 (±108)	2507 (±1079)
**Type III**	30	0	25	4	1	34 (±18)	214 (±92)	2558 (±1068)

BVM: bag-valve-mask; ETT: endotracheal tube; SGA: supraglottic airway; NA: not available

### Ventilation detection performance

Table 2 shows the performance of the ventilation detection algorithm for the test set before and after filtering. For the whole test set, Se/PPV improved from 96.4%/95.0% before filtering to values above 98.2%/97.7%. The results for the clean subset stayed stable before and after filtering. In the distorted subset, Se/PPV improved from 91.9%/89.5% before filtering to values above 97.0%/96.5%. The improvement was much higher for type III records, for which Se/PPV improved from 77.6%/73.5% to values above 95.5%/94.5%.

**Table 2:** Performance of the ventilation detection algorithm before and after filtering for each type of artifact.

Group	Episodes	Before		Fixed-coefficient		Open-loop		Closed-loop	
		Se(%)	PPV(%)	Se(%)	PPV(%)	Se(%)	PPV(%)	Se(%)	PPV(%)
**Total**	202	96.4	95.0	98.4	97.7	98.5	97.9	98.2	98.3
**Clean**	119	99.6	99.0	99.0	98.5	99.2	98.7	99.1	99.2
**Distorted**	83	91.9	89.5	97.7	96.5	97.6	96.7	97.0	97.1
**Type I**	42	97.6	96.2	98.3	97.2	98.3	97.1	98.0	97.6
**Type II**	16	98.5	97.2	98.2	97.7	98.1	98.0	96.5	98.1
**Type III**	25	77.6	73.5	96.3	94.5	96.0	95.1	95.5	95.5

**Fig 6:**
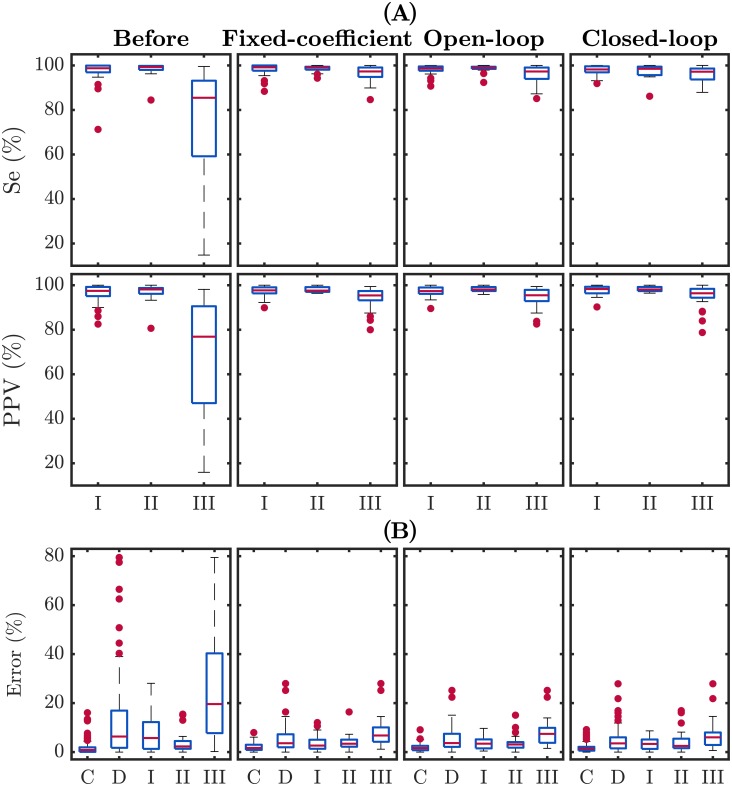
**(A) Distributions of Se/PPV values per episode in each artifact category, before and after filtering. (B) Distribution of the unsigned error in percentage in the estimation of ventilation rate.** Results are provided for all categories: C: clean. D: distorted. I: type I artifact. II: type II; III: type III.

The box plots in Fig 6A show the distribution of Se and PPV per episode for each type of artifact, before and after filtering with the three proposed techniques. Box plots graphically show median (central line in the box) and interquartile values (edges of the box), maximum and minimum values (extreme values of the whiskers), and outliers (red dots). In general, Se and PPV improved after filtering. Furthermore, the high dispersion among type III episodes was drastically reduced after artifact cancellation with all three filtering approaches.

### Ventilation rate estimation

Fig 6B shows the distributions of the unsigned error in percentage per episode between the estimated ventilation rate and the gold-standard value. Again, errors for type III subgroup decreased notably after filtering, as well as errors for type I subgroup, although to a much lesser extent.

### Detection of over-ventilation

Table 3 shows the influence of filtering in the detection of over-ventilation (ventilation rate above 10 min^-1^). From the annotations of the whole dataset, there was a 56.4% (17 901/31 760) of 1-minute intervals with over-ventilation. Globally, the algorithm yielded a Se/PPV of 99.1%/92.6% before and above 97.9%/97.2% after filtering. For the distorted subset, Se/PPV was 98.2%/85.8% before and above 96.3%/95.2% after filtering. Improvement was higher for type III episode, with Se/PPV of 95.5%/72.1% before and above 94.8%/91.1% after filtering.

**Table 3:** Detection of over-ventilation (ventilation rate >10 min^-1^).

Group	Gold Standard		Before		Fixed-coefficient		Open-loop		Closed-loop	
	n_v_	n_hv_	Se(%)	PPV(%)	Se(%)	PPV(%)	Se(%)	PPV (%)	Se(%)	PPV(%)
**Total**	31 760	17 901	99.1	92.6	98.6	97.3	98.4	97.2	97.9	98.0
**Clean**	17 413	10 511	99.7	98.0	99.1	98.3	99.0	98.4	98.9	98.9
**Distorted**	14 347	7 390	98.2	85.8	97.9	95.6	97.4	95.2	96.3	96.6
**Type I**	7 167	3 398	98.9	90.8	98.9	96.8	98.4	96.4	98.0	97.0
**Type II**	2 826	1 837	99.8	96.6	97.6	98.2	97.2	97.8	95.2	98.3
**Type III**	4 354	2 155	95.5	72.1	96.5	91.5	95.9	91.1	94.8	94.2

n_v_ is the number of annotated ventilation rate values in the gold standard (whole set), and n_hv_ is the number of annotated over-ventilation intervals.

## Discussion

In 2010, Idris et al. observed “chest compression oscillations” in more than 70% OOH capnograms [13]. In a recent study, we reported 42% of distorted capnography tracings during CPR [17]. Among the distorted episodes, artifact appearing in the capnogram plateau (type I) was the most prevalent (48%), followed by artifact spanning from the plateau to the baseline (type III) in 31%, and artifact appearing in the capnogram baseline (type II) in 21% of the episodes.

The nature of the artifact is a sinusoid at the frequency of the chest compressions, with varying amplitude. Our findings are in line with the few studies to date which have reported low ventilation volumes incidental to chest compressions [13, 14]. These volumes, although lower than the anatomical dead space, are sufficient to alter the measurement of the capnogram device.

From a clinical perspective, the presence of chest compression artifact has three important drawbacks: first, it impedes the automated detection of ventilations, causing inaccuracies in the measurement of ventilation rate and false over-ventilation alarms. Moreover, the distorted capnogram tracing is difficult to interpret by clinicians. Measurement of reliable ETCO_2_ values becomes impossible, compromising the analysis of ETCO_2_ trends. In conclusion, chest compression artifact may jeopardize most potential uses of capnography during resuscitation, including CPR quality assessment, detection of restoration of spontaneous circulation and prognosis assessment.

The present study focused on the improvement of automated ventilation detection using filtering techniques to pre-process the raw capnogram before the application of the detector algorithm. All the proposed filter schemes performed similarly, reporting favourable Se and PPV values well above 97% and 96%, respectively, for the distorted episodes. This caused an improvement in the measurement of ventilation rate with errors in median below 3.6%, and over-ventilation detection, with Se and PPV values above 96% and 95%, respectively, for the distorted episodes.

The highest improvement was obtained in type III episodes, the most challenging distortion, with Se/PPV in ventilation detection improving from 78%/74% to values higher than 94%. The detector was designed to detect inspiration and expiration downstrokes in a normal capnogram. In the presence of type I artifact the capnogram remains well-above the baseline, i.e. oscillations do not cause false detections of inspiration onsets. Similarly, in the presence of type II artifact, the value of the distorted CO2 is not high enough to detect the expiration upstroke. On the contrary, type III artifact spanning from the plateau to the baseline strongly decreases the ventilation detection. Consequently, the positive impact of filtering is much better observable in type III episodes. In addition, the few studies addressing the artifact phenomenon showed graphical examples of type III capnograms, highlighting the importance of this confounding effect [13, 15].

The adaptive filters should present a better performance than the fixed coefficient filter since compression rates tend to vary during CPR. However, none of the approaches showed a distinctive superiority in terms of performance. The main reason is that, in our recordings, chest compression rate is generally ten times greater than ventilation rate. In this scenario the fixed coefficient filter shows a good performance. The adaptive approaches would be more efficient in case of an excess of ventilation rate with low compression rates. Hence, selection of the filtering algorithm could be analyzed in terms of complexity and computational burden. In this case, adaptive filtering is at a disadvantage compared to the simplicity of a fixed-coefficient filter. Consequently, it seems adequate to apply a filter with fixed coefficients to suppress the chest compression artifact from the capnogram. Nevertheless, the implementation of the three filtering approaches would operate the capnogram signal in real time, being transparent to the user.

The capnogram waveform achieved after filtering approximates the mean peak-to-peak amplitude of the artifact, as illustrated in Fig 5 in the Methods section). After filtering, the capnogram is still difficult to interpret by clinicians. The filtered capnogram waveform hinders the reliable analysis of ETCO_2_ trends, a very useful clinical information during CPR. In the figure, reliable ETCO2 values could only be measured in the undistorted tracing before and after the distorted interval. In practice, capnogram filtering would be an intermediate stage in the ventilation detection algorithm if implemented in the monitor-defibrillator but the resulting waveform would not be displayed on the screen, the raw capnogram would appear instead. The development of other techniques aimed at removing the artifact (to improve ventilation tracking) and at the same time preserving the capnogram tracing would favor clinical interpretation.

## Conclusion

We assessed three filtering alternatives for suppressing the artifact caused by chest compressions on OOH capnograms and analyzed their performance in terms of the improvement of the automated detection of ventilations during CPR. All approaches yielded good results, so simplicity and low computational burden could determine the best alternative to be implemented.

## Supporting information

S1 Appendix Description of the adaptive filters. (PDF)

S1 File Results of the ventilation detection algorithm before and after capnogram filtering. For all episodes in the database, the file contains all ventilations annotated in the gold standard (named with an ordinal number) and ventilation instances detected by the algorithm before and after filtering. Label FP means False Positive detection. Label FN means False Negative detection.
(XLSX)
