# Supplementary material for: Enhancing ventilation detection during cardiopulmonary resuscitation by filtering chest compression artifact from the capnography waveform
Source: PLoS One. 2018 Aug 2;13(8):e0201565. doi: 10.1371/journal.pone.0201565 (PMC6072040; doi:10.1371/journal.pone.0201565)
Supplement: S1 Appendix — (PDF) [file pone.0201565.s001.pdf]

S1 Appendix: Adaptive filtering

Variability of chest compression and ventilation rates during cardiopulmonary resuscitation (CPR) may affect the efficacy of the fixed-coefficient filter [1–4]. Adaptive techniques in which the filter parameters are adjusted in time according to the varying characteristics of the artifact could be a suitable solution. In the literature, adaptive filtering has been extensively used for the suppression of the artifact induced by chest compressions in the electrocardiogram recorded by defibrillators during CPR [5–9].

For the capnogram artifact issue, we designed two different adaptive filtering configurations, an open-loop and a closed-loop adaptive filter [10].

Open-loop (OL) adaptive filter:

The OL adaptation is illustrated in Figure 1A. This technique is based on the adaptive adjustment of the filter to the information extracted from the compression depth (CD) signal, used as a reference in this case.

The filter was a stop-band Butterworth filter whose central frequency is adaptively adjusted according to the chest compression rate. We estimated the average chest compression rate in 2-s intervals, using the annotated compression instances in the CD signal. Thus, the coefficients of the filter were updated every 2 s [10].

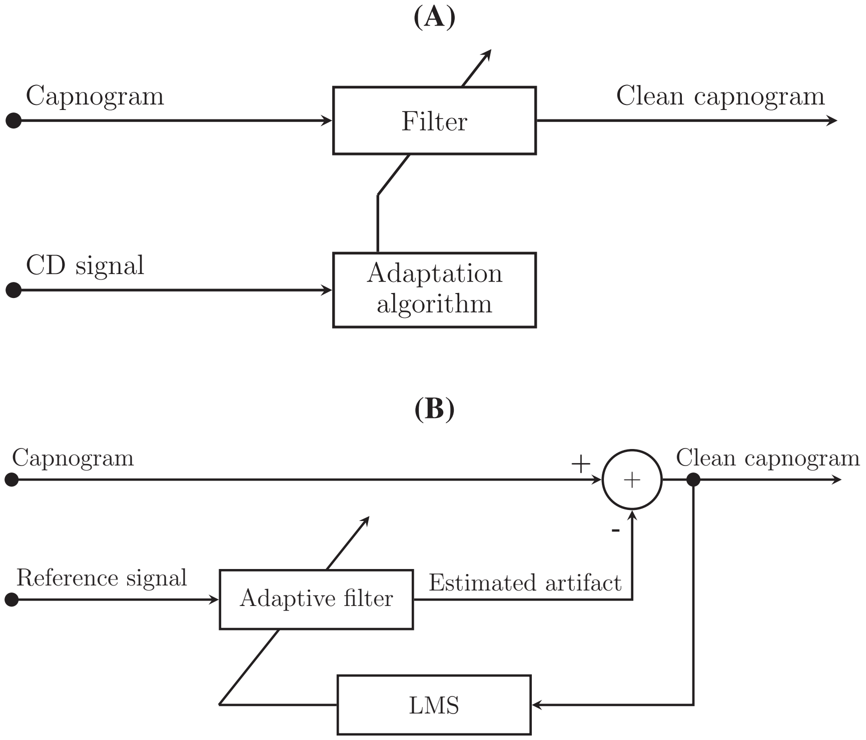

Figure 1: **(A) Open-loop adaptive filter.** The adaptation algorithm uses the capnogram and the CD signal to adjust the filter coefficients every 2-s. **(B) Closed-loop adaptive filter.** Configuration for adaptive cancellation of the chest compression artifact.

Closed-loop (CL) adaptive filter:

Figure 1B shows the scheme of the CL adaptive filter applied to canceling sinusoidal interference [10]. This system requires an additional reference input signal. In our approach, this reference signal is modeled as a pure cosine wave of time-varying amplitude and phase, with a fundamental frequency matching the chest compression rate. The chest compression instants detected in the CD signal were used to estimate the instantaneous chest compression rate, which may vary between compressions.

In this configuration, the artifact is adaptively estimated and subtracted from the input capnogram to obtain a clean capnogram. The filter coefficients are updated sample by sample by applying the Least-Mean-Square algorithm (LMS). The resulting system is equivalent to a notch filter at the frequency of chest compressions, capable of adaptively tracking the exact frequency of the artifact [10].

References

1. Abella BS, Sandbo N, Vassilatos P, Alvarado JP, O'hearn N, Wigder HN, Becker LB. Chest compression rates during cardiopulmonary resuscitation are suboptimal: a prospective study during in-hospital cardiac arrest. Circulation. 2005;111(4):428–434.

2. Meaney PA, Bobrow BJ, Mancini ME, Christenson J, De Caen AR, Bhanji F, Aufderheide TP. Cardiopulmonary resuscitation quality: improving cardiac resuscitation outcomes both inside and outside the hospital: a consensus statement from the American Heart Association. Circulation. 2013;128(4):417–435.

3. O'Neill JF, Deakin CD. Do we hyperventilate cardiac arrest patients? Resuscitation. 2007;73(1):82–85.

4. Maertens VL, De Smedt LE, Lemoyne S, et al. Patients with cardiac arrest are ventilated two times faster than guidelines recommend: an observational prehospital study using tracheal pressure measurement. Resuscitation. 2013;84(7):921–926.

5. Aase SO, Eftestol T, Husoy J, Sunde K, Steen PA. CPR artifact removal from human ECG using optimal multichannel filtering. IEEE Trans Biomed Eng. 2000;47(11):1440–1449.

6. Eilevstjønn J, Eftestøl T, Aase SO, Myklebust H, Husøy JH, Steen PA. Feasibility of shock advice analysis during CPR through removal of CPR artefacts from the human ECG. Resuscitation. 2004;61(2):131–141.

7. Irusta U, Ruiz J, de Gauna SR, Eftestøl T, Kramer-Johansen J. A least mean-square filter for the estimation of the cardiopulmonary resuscitation artifact based on the frequency of the compressions. IEEE Trans Biomed Eng. 2009;56(4):1052–1062.

8. Gong Y, Chen B, Li Y. A review of the performance of artifact filtering algorithms for cardiopulmonary resuscitation. J Healthc Eng. 2013;4(2):185–202.

9. Ruiz de Gauna S, Irusta U, Ruiz J, Ayala U, Aramendi E, Eftestøl T. Rhythm analysis during cardiopulmonary resuscitation: past, present, and future. Biomed Res Int. 2014;2014.

10. Widrow B, Stearns SD. Adaptive signal processing. Englewood Cliffs, NJ, Prentice-Hall, Inc. 1985;1:491.
